# Collective single-photon emission and energy transfer in thin-layer dielectric and plasmonic systems

**DOI:** 10.1515/nanoph-2024-0524

**Published:** 2025-01-30

**Authors:** Mads A. Jørgensen, Devashish Pandey, Ehsan Amooghorban, Sanshui Xiao, Nicolas Stenger, Martijn Wubs

**Affiliations:** Department of Electrical and Photonics Engineering, Technical University of Denmark, Kgs. Lyngby, Denmark; Department of Physics, Faculty of Science, Shahrekord University, P.O. Box 115, Shahrekord 88186-34141, Iran; Center for Nanostructured Graphene, Technical University of Denmark, Kgs. Lyngby, Denmark; NanoPhoton – Center for Nanophotonics, Technical University of Denmark, Kgs. Lyngby, Denmark

**Keywords:** superradiance, FRET, local density of optical states, cross density of optical states, hexagonal boron nitride, surface plasmon polariton

## Abstract

We study the collective photon decay of multiple quantum emitters embedded in a thin high-index dielectric layer such as hexagonal boron nitride (hBN), with and without a metal substrate. We first explore the significant role that guided modes including surface plasmon modes play in the collective decay of identical single-photon emitters (super- and subradiance). Surprisingly, on distances relevant for collective emission, the guided or surface-plasmon modes do not always enhance the collective emission. We identify configurations with inhibition, and others with enhancement of the dipole interaction due to the guided modes. We interpret our results in terms of local and cross densities of optical states. In the same structure, we show a remarkably favorable configuration for enhanced Förster resonance energy transfer between a donor and acceptor in the dielectric layer on a metallic substrate. We compare our results to theoretical limits for energy transfer efficiency.

## 1 Introduction

The cooperative emission from multiple emitters is the foundation of promising new technologies like superradiant lasers [1], [2], [3], [4], [5], single-photon emission [6] or quantum memory for information processing [7]. Super- and subradiance are the respectively faster or slower emission of light from multiple emitters due to a shared coupling to the electromagnetic environment. It was originally described in a many-emitter, many-photon limit in free space [8], [9], [10], [11], [12], [13]. For multi-emitter technology, it is also important to study another limit of collective emission, namely a single photon shared between few emitters [14], [15], [16], in an engineered photonic environment.

Superradiance in solid state systems is an active field of study [17], [18], [19], [20], [21], [22] and often involves waveguide geometries [23], [24], [25], [26], [27]. Here we will consider quantum emitters in Van der Waals materials or subwavelength thin films, which by themselves already are planar waveguides. For distant emitters along one-dimensional waveguides, waveguide modes dominate the collective emission [24], [27], but for our planar waveguides the situation is less clear.

We study how collective emission could be enhanced or suppressed by embedding the thin film that contains the quantum light sources into other planar photonic waveguide structures. As our main example, we will consider strongly subwavelength-thin films of hexagonal boron nitride (hBN), which has a large band gap of around 6 eV and can host various types of single-photon emitting color centers [28], [29], [30], [31], [32], [33], [34]. Some of these have narrow emission lines even at room temperature [28] and can be deterministically localized [35].

Well known challenges for observing collective emission in solid-state environments include inhomogeneous broadening, spectral diffusion, and decoherence due to phonon interactions [22], [36]. Nevertheless, collective light emission has been observed in various solid-state environments [24], [27], [36], [37]. Decoherence due to phonons can be reduced by working at lower temperatures, although cryogenic temperatures by themselves are no guarantee for lifetime-limited operation of color centers in hBN [38]. Recently electric-field modulation was shown to actively reduce spectral diffusion and to tune emission lines and narrow linewidths of color centers in hBN down to almost the homogeneous lifetime linewith [39]. This may also bring closer the observation of collective emission by color centers in hBN. Other promising emitters in hBN are so-called blue color centers, which can be produced deterministically by electron beams and show surprisingly little inhomogeneous broadening [32].

Inspired by these developments, we study how collective emission of single photons can be enhanced or suppressed among resonant lifetime-limited color centers in hBN, by engineering their interaction. Besides this idealized coherent limit, where collective emission takes place, we will also consider the opposite incoherent limit, where energy exchange between embedded emitters is a one-way energy transfer process.

In both limits, we ask how the layered environment, with its associated guided modes, affects the interaction between emitters.

**Figure 1:**
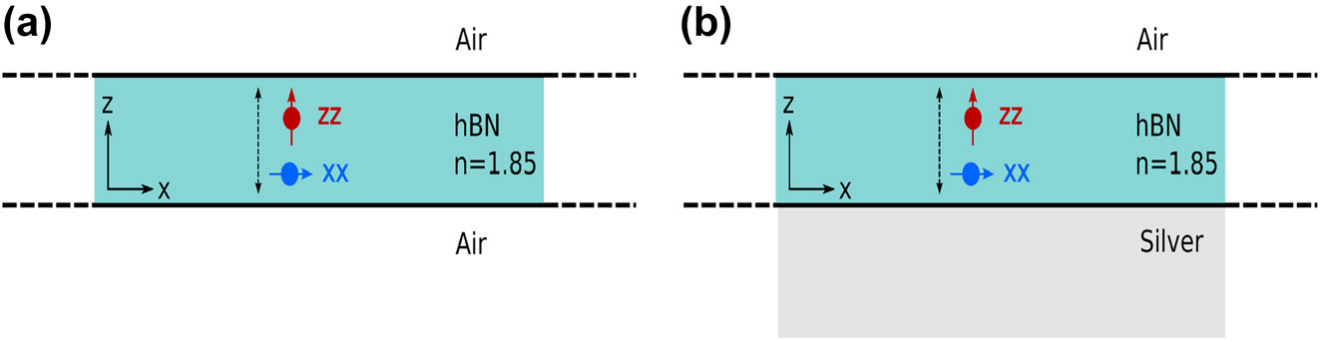
A thin layer of hBN, of thickness *d* = *λ*/10, is surrounded by air on top and by (a) air or (b) silver below. One or more emitters are embedded in the hBN layer. We consider both in-plane and out-of-plane dipole orientations, both corresponding to reported types of emitters in hBN.

Metal interfaces and metallic nano-particles are widely used to enhance light–matter interactions by making use of their associated surface plasmons [40], [41], [42], [43], [44], [45]. Here we report how interactions between embedded quantum emitters are influenced by positioning the thin film onto a metal substrate, as sketched in Figure 1.

We will also consider the role of dipole orientations; usually dipoles in 2D materials point in the plane of the atomic layers, but out of plane dipoles have also been reported [46], [47].

In Section 2 we introduce basics of light emission in terms of the classical Green function, and how to calculate the latter for a multilayer medium. In Section 3 we calculate single-emitter decay rates, inter-emitter interactions, and collective emission rates. We isolate the contribution of the guided modes to the total interaction and find both enhanced and suppressed inter-emitter interaction due to the interference of guided modes with the radiative modes. We interpret these results in terms of the concepts of local density of optical states (LDOS) and cross density of optical states (CDOS). Subsequently, we calculate super- and subradiant decay rates for emitters in the top of the hBN layer and find both enhanced collective decay at finite emitter separations as well as a longer range for collective emission. For the same multilayer geometry, we also study energy transfer rates (incoherent limit) that are governed by the same classical Green function, and we find a donor-acceptor configuration with a surprisingly efficient resonant energy transfer. We compare this with theoretical limits for the energy transfer efficiency. We end with a discussion and conclusions.

## 2 Emission, transfer, and green function

In this section we introduce the important concepts needed to analyze spontaneous and collective emission, as well as energy transfer in the layered media that we will consider.

*Single-atom decay rates* and local densities of optical states (LDOS) have been calculated for layered systems before [40], [48], [49], [50]. The decay of an emitter depends on its electromagnetic environment and the response to light of any given medium is encoded in the corresponding classical electromagnetic Green function $\overset{⃡}{G}$ (a tensor) of the medium, defined by.

(1) $$\left(\epsilon \left(r,\omega \right)\frac{\omega^{2}}{c^{2}}\text{I}-\nabla \times \nabla \;\times \right)\overset{⃡}{G}\left(r,r^{\prime },\omega \right)=\delta \left(r-r^{\prime }\right)\text{I},$$

where *ϵ*(**r**, *ω*) is the inhomogeneous dielectric function (here assumed scalar) and I is the 3 × 3 identity matrix. The spontaneous-decay rate Γ of an emitter with dipole moment ***μ*** at position **r** radiating at frequency *ω* is given by

(2) $$\Gamma =2\gamma =-\frac{2\mu^{2}\omega^{2}}{\hbar \epsilon_{0}c^{2}}\hat{\mu }\cdot \text{Im}\left[\overset{⃡}{G}\left(r,r,\omega \right)\right]\cdot \hat{\mu },$$

in terms of the imaginary part of the Green function. This is a self-interaction in the sense that the Green function is twice evaluated at the same spatial point **r**, in our case a point within the hBN layer of Figure 1. This intensity decay rate Γ equals twice the field amplitude decay rate *γ* and is proportional to the partial LDOS [51], [52] (here called PLDOS):

(3) $$\begin{array}{ll} \text{PLDOS} & =-\frac{6\omega }{\pi c^{2}}\hat{\mu }\cdot \text{Im}\left[\overset{⃡}{G}\left(r,r,\omega \right)\right]\cdot \hat{\mu }\\ & =3\underset{\lambda }{\sum }{\left|f_{\lambda }\left(r\right)\cdot \hat{\mu }\right|}^{2}\delta \left(\omega -\omega_{\lambda }\right). \end{array}$$

It follows immediately that Γ = (*μ* ^2^ *ωπ*/(3*ℏɛ*_0_))PLDOS. This PLDOS would turn into the full LDOS by averaging over dipole orientations, but we will not do that here since we consider fixed dipole orientations. The last equality in Eq. (3) features a sum over a complete set of optical eigenmodes **f**_*λ*_(**r**) of the dielectric environment, and shows explicitly that the PLDOS is a non-negative quantity. Such a complete eigenmode expansion is only possible for real-valued non-dispersive dielectric functions.

A number *N* of quantum emitters does not always emit light at their respective single-atom decay rates, however. Collective emission can take place if the emitters are (nearly) resonant and their interactions strong enough to overcome both detuning and dephasing. The latter requires them to be either in each other’s near field and/or they should be connected by a waveguide. The decay dynamics will then be described by *N* collective decay rates that depend both on the single-emitter decay rates, the detunings between the emitters, and on the pairwise interaction strengths between the emitters. The emitter-emitter interaction is described by the full classical Green function (i.e. both its real and imaginary parts) that connects the locations of the pair of emitters. In an inhomogeneous nanophotonic environment it is not necessarily so that all emitters have equal transition frequencies and equal single-atom decay rates. But if this is the case, then the collective decay rates that are larger (smaller) than the single-atom decay rate can be called superradiant (subradiant), analogous to the naming for free space.

Therefore, in order to determine *collective emission rates*, we need to quantify the full interaction, of emitters in a complex environment. This is more challenging than calculating the PLDOS, due to the highly singular behaviour of the real part of the Green function in the near field. Spatially separated emitters can interact via a shared coupling to the electromagnetic environment. Using a multiple-scattering formalism that takes into account all scattering events between any number of emitters [16], [53], or a master-equation formalism [22], [54], [55], the collective modes of two quantum emitters in a photonic environment can again be expressed in terms of the Green’s function $\overset{⃡}{G}$ of that environment.

So that is how we will calculate the interaction between dipoles in the thin films, recall Figure 1. We will make the simplifying assumptions of two identical and lifetime-limited emitters, positioned at **r**_1_ and **r**_2_ at equal heights. This equal-height assumption is good for color centers generated by irradiation as in Refs. [30], [31]. With these assumptions, their single-atom emission rates become identical. (In practice, transition frequencies of two emitters will differ and need external tuning to be brought into resonance.) For such a pair of quantum emitters, the complex frequencies of the super- and subradiant modes are found as

(4) $$\omega^{\pm }=\tilde{\Omega }-i\gamma \pm J_{12}\left(\tilde{\Omega }\right),$$

where

(5) $$J_{12}\left(\omega \right)=\frac{\mu^{2}\omega^{2}}{\hbar \epsilon_{0}c^{2}}{\hat{\mu }}_{1}\cdot \overset{⃡}{G}\left(r_{1},r_{2},\omega \right)\cdot {\hat{\mu }}_{2}$$

is the complex-valued inter-emitter interaction and $\tilde{\Omega }$ is the phenomenologically observable emission frequency into which the self-interaction has been absorbed. The generalization of Eq. (4) to detuned transition frequencies ${\tilde{\Omega }}_{1}\neq {\tilde{\Omega }}_{2}$ and/or unequal single-atom emission rates *γ*_1_ ≠ *γ*_2_ wil not be considered here but can be found in Refs. [16], [27], [53].

The electromagnetic Green’s function appears as the mediator of the electromagnetic field. It encodes all of the information about the response of the environment in which the emitters are treated as scatterers. From Eq. (4) it is clear that the real and imaginary parts of *J*_12_ play fundamentally different roles: the real part constitutes a shift in emission frequency, sometimes called the collective Lamb shift, while the imaginary part of *J*_12_ influences the two collective decay rates

(6) $$\gamma^{\pm }=-\text{Im}\left[\omega^{\pm }\right]=\gamma \mp \text{Im}\left[J_{12}\right].$$

Here the interaction *J*_12_ is not a self-interaction, i.e. the Green function in Eq. (5) describes the propagation of light between two different emitters. Its imaginary part $Im\left[\overset{⃡}{G}\left(r_{1},r_{2},\omega \right)\right]$ is proportional to the partial cross density of optical states, or partial CDOS [18], here further abbreviated as PCDOS,

(7) $$PCDOS=-\frac{6\omega }{\pi c^{2}}\hat{\mu_{1}}\cdot \text{Im}\left[\overset{⃡}{G}\left(r_{1},r_{2},\omega \right)\right]\cdot \hat{\mu_{2}},$$

which is a measure of the number of electromagnetic modes connecting two points, per energy [18], [19], and becomes a PLDOS if the two positions and dipole moments become equal. Just like the PLDOS, the PCDOS can be expanded into a sum of a complete set of optical modes [56],

(8) $$\text{PCDOS}=3\underset{\lambda }{\sum }{\hat{\mu }}_{1}\cdot \text{Re}\left[f_{\lambda }\left(r_{1}\right)f_{\lambda }^{*}\left(r_{2}\right)\right]\cdot {\hat{\mu }}_{2}\delta \left(\omega -\omega_{\lambda }\right).$$

So the PCDOS adds up how all the modes connect two unit vectors ${\hat{\mu }}_{1,2}$ in two points **r**_1,2_. The PCDOS plays an important role in the collective decay of two emitters with identical observable emission frequencies, and satisfies the identity $\text{PCDOS}=-6\hbar \varepsilon_{0}/\left(\mu^{2}\omega \pi \right)\text{Im}\;\left[J_{12}\right]$ .

Super- and subradiance can occur when quantum coherences survive on the time scale of spontaneous emission. In the opposite incoherent limit, energy transfer between emitters can still take place, by Förster resonance energy transfer (FRET). FRET is the process of energy transfer between two particles through the exchange of virtual photons in the near-field of the emitters. Typical inter-emitter separations are a few nanometers [57], [58], [59], [60]. FRET goes from an initially excited donor emitter to a lower-energy acceptor, with broad overlapping emission and absorption spectra.

The classical Green function describes not only superradiance but also the one-way FRET rate Γ_FRET_ from the donor (D) to the acceptor (A), as [61]

(9) $$\Gamma_{\text{FRET}}=\overset{\infty }{\underset{-\infty }{\int }}\text{d}\omega \;\sigma_{\text{DA}}\left(\omega \right)|J_{\text{DA}}\left(\omega ,r_{\text{A}},r_{\text{D}}\right){|}^{2}.$$

Here the spectral overlap function *σ*_DA_(*ω*) is proportional to the product of the donor emission spectum *σ*_D_(*ω*) and the acceptor absorption spectrum *σ*_A_(*ω*). In general, there is no direct proportionality between the LDOS and the FRET rate [62], [63].

An excited donor emitter either emits spontaneously or transfers its photon energy to the acceptor. The corresponding FRET efficiency *η*_FRET_ is given by

(10) $$\eta_{\text{FRET}}=\frac{\Gamma_{\text{FRET}}}{\Gamma_{\text{FRET}}+\Gamma^{D}}=\frac{\Gamma_{\text{FRET}}}{\Gamma_{\text{FRET}}+\Gamma_{\text{rad}}^{D}+\Gamma_{\text{nrad}}^{D}},$$

where Γ_FRET_ is the total FRET transfer rate between the donor and acceptor, and $\Gamma^{D}=\Gamma_{\text{rad}}^{D}+\Gamma_{\text{nrad}}^{D}$ is the total decay rate of the donor, including the radiative and non-radiative decay rates, respectively. For comparison, the fluorescence quantum yield of the donor is obtained from Eq. (10) by replacing Γ_FRET_ in the numerator by $\Gamma_{\text{rad}}^{D}$ , which shows that FRET efficiency and quantum yield are efficiencies of competing processes that add up to unity if $\Gamma_{\text{nrad}}^{D}=0$ (and note that $\Gamma_{\text{nrad}}^{D}$ does not contain Γ_FRET_ in our definition).

Eq. (10) for the FRET efficiency is only valid in the limit where the excitation jumping back from the acceptor to the donor can be neglected, that is when Γ^*A*^ ≫Γ_FRET_, Γ^*D*^. If the transfer is to be efficient, the interaction has to be large enough to compete with single-emitter decay of the donor. Both the FRET rate and the FRET efficiency increase if the spectral overlap of the donor and acceptor emitters grows. Below we propose instead a specific geometry that presents an alternative way to increase the FRET efficiency in Eq. (10), namely by suppressing the decay rate of the donor while keeping the total FRET rate high.

## 3 Results

In the following, we will compare two three-layer structures, the first one consisting of a thin layer of hBN (*ϵ*_hBN_ = 1.85^2^ = 3.4225 and thickness *d* = *λ*/10 [64]) in air (*ϵ*_air_ = 1). The second structure consists of the same thin hBN layer now placed in between air and a silver substrate with *ϵ*_Ag_ = −13.529 + 0.416*i* [65]. These dielectric constants are tabulated values at *λ* = 560 nm, since hBN can host bright quantum emitters at this wavelength with a corresponding photon energy of 2.2 eV [28], [31], [34].

We will calculate position- and frequency-dependent single-emitter decay rates described by Eq. (7), collective decay rates as in Eq. (4), and energy-transfer rates according to Eq. (9). The unknowns in these equations are related to the classical Green function for the layered systems considered. We will calculate these Green functions as integrals over in-plane wavevectors, relegating most technical details to appendices, and separate out the effects of guided and surface-plasmon modes from the other electromagnetic modes.

### 3.1 Single-emitter decay in a thin slab

**Figure 2:**
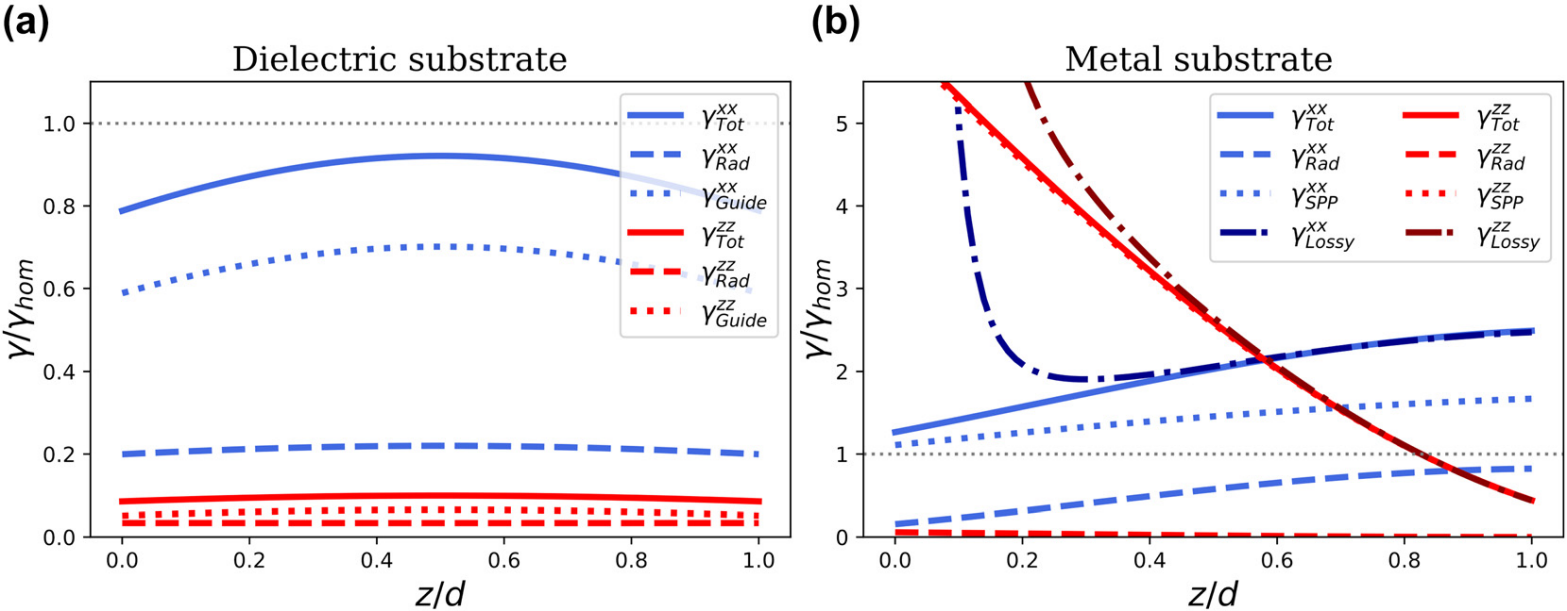
Decay rate of a single emitter at a wavelength *λ* = 560 nm as a function of scaled height *z*/*d* in an hBN layer of thickness *d* = *λ*/10 for (a) an air/hBN/air and (b) an air/hBN/silver three-layer system. The value *z* = *d* corresponds to the top hBN-air interface. Blue (red) curves correspond to in-plane (out-of-plane) dipoles. The total decay rates are shown in solid lines, while combined contributions of guided and SPP modes are shown in dotted lines. The differences between solid and dotted lines are shown as dashed lines and labeled as “radiative” contributions. In (b), the dark blue and dark red dash-dotted lines are total decay rates for silver (*ϵ* = −13.529 + 0.416*i*), while the light blue and bright red curves correspond to “lossless silver” (with real-valued *ϵ* = −13.529).

Before studying collective emission in multilayers, for comparison we first study single-atom emission in the same geometries. So we first consider a lifetime-limited single dipole emitter embedded inside the central hBN layer. Its decay rate will be orientation-dependent and the three-layer Green’s function that is to be inserted into Eq. (7) will reflect this. We will only consider fully in-plane or fully out-of-plane dipole orientations. Both of these high-symmetry cases have been observed for color centers in hBN [46], [66]. Figure 2(a) shows the spontaneous-decay rate of an emitter inside the hBN layer as a function of the emitter’s height within the layer, both for in-plane and for out-of-plane dipole orientations, and normalized by the decay rate in homogeneous hBN. The guided-mode contribution *γ*_Guide_ is shown in dotted lines and includes the sum of both the TE and TM modes, which are found at mode indices (defined in App. A.1) $\alpha_{\text{Guide}}^{\text{TE}}=1.201$ and $\alpha_{\text{Guide}}^{\text{TM}}=1.027$ , respectively. An important role in the decay of the emitter is played by the guided modes, with their combined rate *γ*_Guide_ typically larger than the rate *γ*_rad_ corresponding to the remaining radiative decay channels. The total single-emitter decay rate *γ*_Tot_ is given by the sum of *γ*_Guide_ and *γ*_Rad_. For simplicity, we do not consider decay through other non-radiative channels, i.e. not described by the complex dielectric function *ɛ*, that may reduce the quantum efficiency of the emitters further.

Figure 2(a) shows a strong suppression of emission by the out-of-plane oriented dipole, which emits only about one-tenth as frequently as the same emitter in the homogeneous medium. The situation is different when the thin film rests on a metal substrate. The decay rate of an emitter in hBN on top of silver, with and without losses, is shown in Figure 2(b). The presence of the SPP mode (*α* = 1.69) enhances the light–matter interaction, resulting in decay rates that are large compared to those of the homogeneous hBN. The relative contribution of the SPP mode is weakened with increasing distance to the metal interface for the in-plane dipole orientation, but for the out-of-plane dipole, the SPP mode in lossless silver is responsible for nearly all of the decay. The lossy metal provides a means of single-emitter decay through Ohmic losses for the emitter when it is close. For a Drude metal this energy transfer to the metal occurs at a rate ∝ *f*(*ω*)/*d* ^3^, with a frequency-dependent coefficient *f*(*ω*) that peaks at the surface-plasmon resonance energy of the interface, but also away from this resonance results in a diverging decay rate as the emitter approaches the metal ”quenching”) [67]. However, the Ohmic losses become negligible compared to the other means of decay already for distances beyond a few nanometers between emitter and metal interface.

### 3.2 Interactions between two resonant emitters

**Figure 3:**
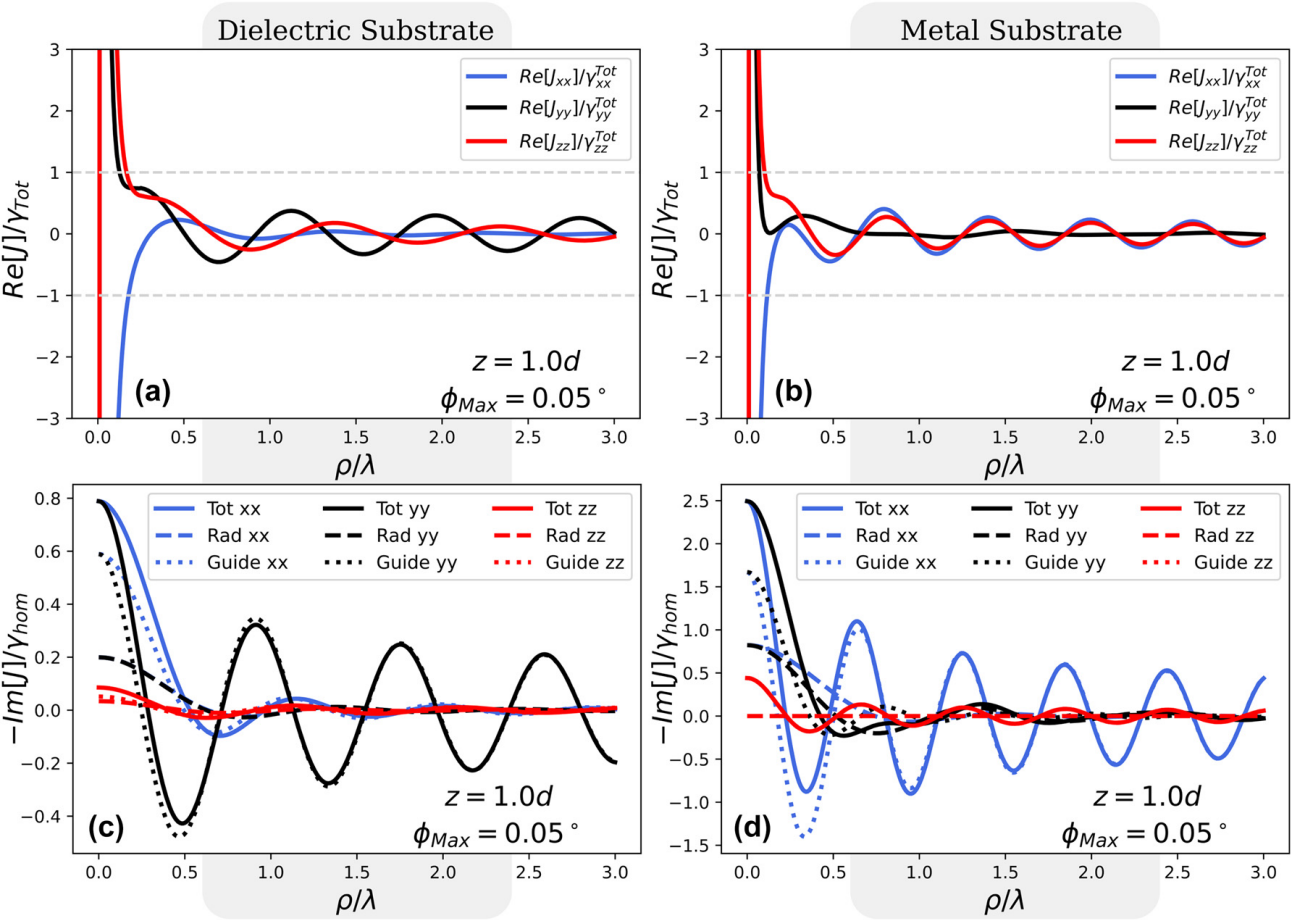
Real and imaginary parts of the interaction *J*_12_ between two emitters placed at the top of a central hBN (*ϵ*_hBN_ = 3.4225) layer of thickness *d* = *λ*/10, as a function of their separation along the *x*-direction. An air substrate is used in panels (a) and (c) while a (lossless) silver substrate (*ϵ*_LS_ = −13.529) is used in (b) and (d) in order to determine the SPP contribution. In the legend on the right, the total imaginary parts of the interaction are shown in solid while the contributions from the guided modes are shown in dotted lines. The remaining contribution, shown as dashed, is labeled ’rad’. The normalization in (a) and (b) is the position- and orientation-dependent decay rate of the single emitter in the layered medium, while (c) and (d) are normalized by the single-emitter decay rate in bulk hBN as shown in Figure 2. The angle *ϕ*_max_ is related to the numerical calculation as explained in App. C.

We consider two emitters in the central layer of hBN, each with dipole moment ***μ***_*m*_ interacting via a single frequency *ω* as in Eq. (5). Color centers in hBN produced by ion irradiation tend to exist in the uppermost layers [30], [31], and here we will limit ourselves to this case where the emitters are placed at the very top of the hBN layer, *z* ≈ *z*_0_ = *d*, furthest from the substrate, whereby we can also model molecular emitters adsorbed on the surface of the hBN substrate [68], [69]. There have been reports of dipole orientations both in the plane [66] and out-of-plane [46], [47]. Figure 3 shows the real and imaginary parts of the interaction *J*_12_ between two identical emitters with parallel dipole moments, as a function of the separation along the *ρ* = *x*-axis. So we define the *x*-direction to point along the line joining the two emitters, the *y*-direction is the in-plane direction perpendicular to *x*, and *z* points out of plane.

In order to realize collective emission, the emitter-emitter interaction rate has to become of the order of the single-emitter decay rate. In experiments, the interaction also needs to overcome single-atom dephasing and non-radiative decay rates, if any, both of which would lead to broadening of the emission spectrum, but we do not consider these additional challenges here.

If one wishes to spectrally resolve the split-peak character of the super- and subradiant modes due to the collective Lamb shift in Eq. (4), an effect which is characteristic of strongly coupled systems, a Rayleigh criterion of |Re[*J*_12_]| > *γ*_tot_ requires the emitters to be in the near-field, as shown in Figure 3(a) and (b). In these two panels (a) and (b) we have made the less common choice of normalizing with the position- and orientation-dependent decay rate, because it allows easy graphical inspection of when the Rayleigh criterion holds. This tells us at what distances collective emission of lifetime-limited emitters is feasible for the configuration studied. The equality |Re[*J*_12_]| = *γ*_tot_ is marked by the horizontal grey dashed lines. It can be seen that the Rayleigh criterion is satisfied by emitters approximately at a tenth of the wavelength or less.

For Im[*J*_12_] in panels (c) and (d), now scaled by the decay rate of a homogeneous hBN medium, we see that the dielectric system in (c) favours collective emission in the *yy* − orientation, in the sense that the position-dependent oscillations in Im[*J*_12_]/*γ*_hom_ have largest amplitudes for those dipole orientations. This can almost fully be ascribed to the coupling between the emitters via the guided mode (dotted curve) of the hBN slab, already for horizontal distances *ρ* of half a wavelength. The dominance of the waveguide mode at large distances is as expected, while at these short distances it is more surprising. Around *ρ*/*λ* = 0.5 in panel (c), we see that the signs of the guided and radiative parts of Im[*J*_12_] are opposite, resulting in a total Im[*J*_12_] that has a smaller absolute value than its guided part alone. This destructive interference between different channels for interatomic interactions will thus lead to weaker interaction between emitters.

By contrast, the plasmonic system in Figure 3(d) favours collective emission for the in-plane *xx* dipole orientation, despite the fact that also here considerable ‘interaction cancellation’ happens due to sign differences of the guided and radiative parts of Im[*J*_12_], especially for *ρ*/*λ* between 0.2 and 0.5.

With the silver substrate present, there is a range of separations where destructive interference between the guided and radiative modes of the in-plane-oriented emitters cause the total interaction to be suppressed. This is most notable for the *yy* − configuration, and means that plasmons are not always beneficial and can be detrimental, not due to their lossy nature, but due to destructive interference. This being said, the interaction between emitters is dominated by the guided modes, both distant and in the near-field. The long-range interaction is mediated almost entirely by the guided modes, illustrated by the merging of the dotted and solid lines in Figure 3.

### 3.3 Two-emitter superradiance

**Figure 4:**
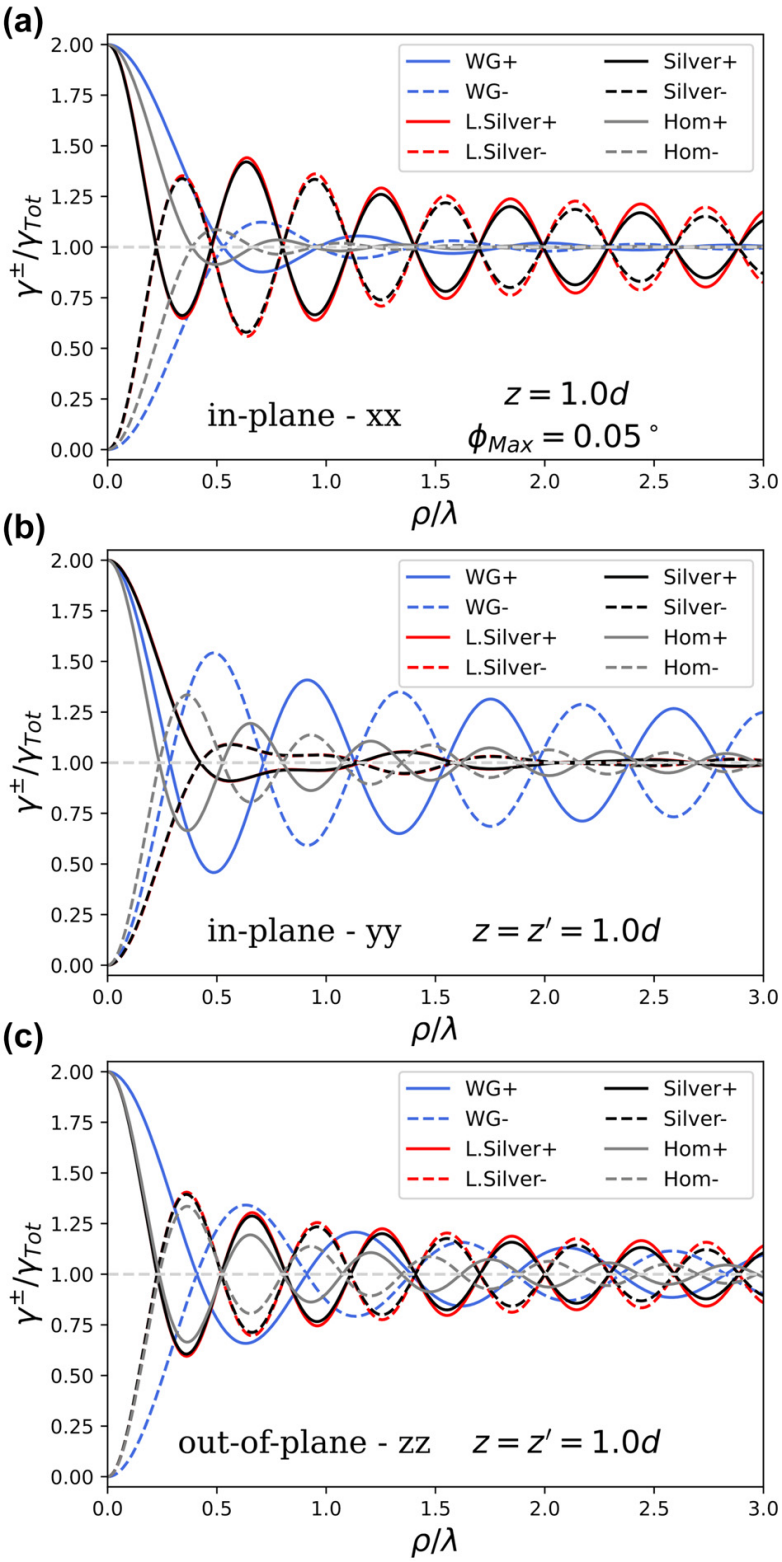
Super- and subradiant decay rates of two identical emitters situated at the top of a thin (*d* = 0.1*λ*) slab of hBN with air above and either air (blue), lossless silver (red) or lossy silver (black) below. These rates are normalized by the corresponding single-emitter decay rates in the given medium for the same dipole orientation. The axis of separation is along $\hat{x}$ and the dipoles are parallel and oriented along (a) $\hat{x}$ , (b) $\hat{y}$ and (c) $\hat{z}$ , respectively. The decay rates corresponding to *γ* ^±^ = −Im[*ω* ^±^] of equation (6) for two emitters are shown in solid (dashed) for plus (minus) modes, respectively. The corresponding collective decay rates of emitters in homogeneous hBN are shown in grey.

The super- and subradiant decay rates of identical emitters are given in Eq. (6) and shown in Figure 4 for emitters at the top of the hBN layer. The dielectric waveguide (blue), lossy (red) and lossless (black) plasmonic systems are compared to the rates for homogeneous hBN (grey). For all orientations the amplitude of the oscillating collective decay rates in the layered medium surpass those of the homogeneous medium, though the difference is largest for in-plane emitters.

Comparing the red and black curves in Figure 4, corresponding to the layered air/hBN/silver system with lossless and lossy silver, respectively, we find that Ohmic losses are mostly negligible for collective decay, at least for emitter separations up to a few optical optical wavelength as shown here. Ohmic losses only really become significant as distances approach the propagation length of surface plasmons, which is on the order of multiple microns [70], [71], or if at least one of the emitters is very close to the metal interface, where its emission may be quenched [67], [72], [73], as we mentioned earlier.

Remarkably, for the *xx* − orientation in Figure 4(a), the collective rates *γ* ^±^ show more pronounced peaks and valleys near *ρ*/*λ* ≈ 0.6 than for the extrema at the shorter distance *ρ*/*λ* ≈ 0.35 (red and black curves). Analogous features occurred in Im[*J*] in Figure 3(d). By contrast, for a bulk medium the analogous collective emission rates for two emitters with equal *z*-coordinates are depicted as the grey curves in Figure 4(a–c), showing that oscillations in the rates at larger distances are more damped. Here the reverse situation can occur for the layered plasmonic medium, because of destructive interference between radiative modes on the one hand and guided modes (including surface plasmons) on the other.

For each in-plane orientation of the emitters, there is one type of layered system that exhibits enhanced collective decay for finite emitter separations; the dielectric substrate favours the *yy*-configuration while the silver substrate favours the *xx*-configuration. On the other hand, in Figure 4(b) for separations *ρ*/*λ* ≳ 0.3 we see very strong suppression of the collective decay rate of emitters in the *yy*-configuration in the plasmonic system, again due to the cancellation of the radiative and guided mode contributions to the PCDOS. This suppression means that this combination of medium and emitter orientation gives less pronounced collective decay than emitters in bulk hBN at the same distances, although the latter have no guided modes.

### 3.4 Plasmon-assisted efficient FRET

Next we examine the one-way Förster energy transfer [57] between two emitters located at different heights within the hBN layer on the silver substrate by utilizing the large differences between the single-emitter decay rates of the donor and the acceptor. We are inspired by Ref. [42] where it is proposed to enhance of FRET with the help of localized surface plasmons in metal nanoparticles. We will instead consider enhancement of the FRET efficiency helped by the propagating SPPs in our planar geometry.

**Figure 5:**
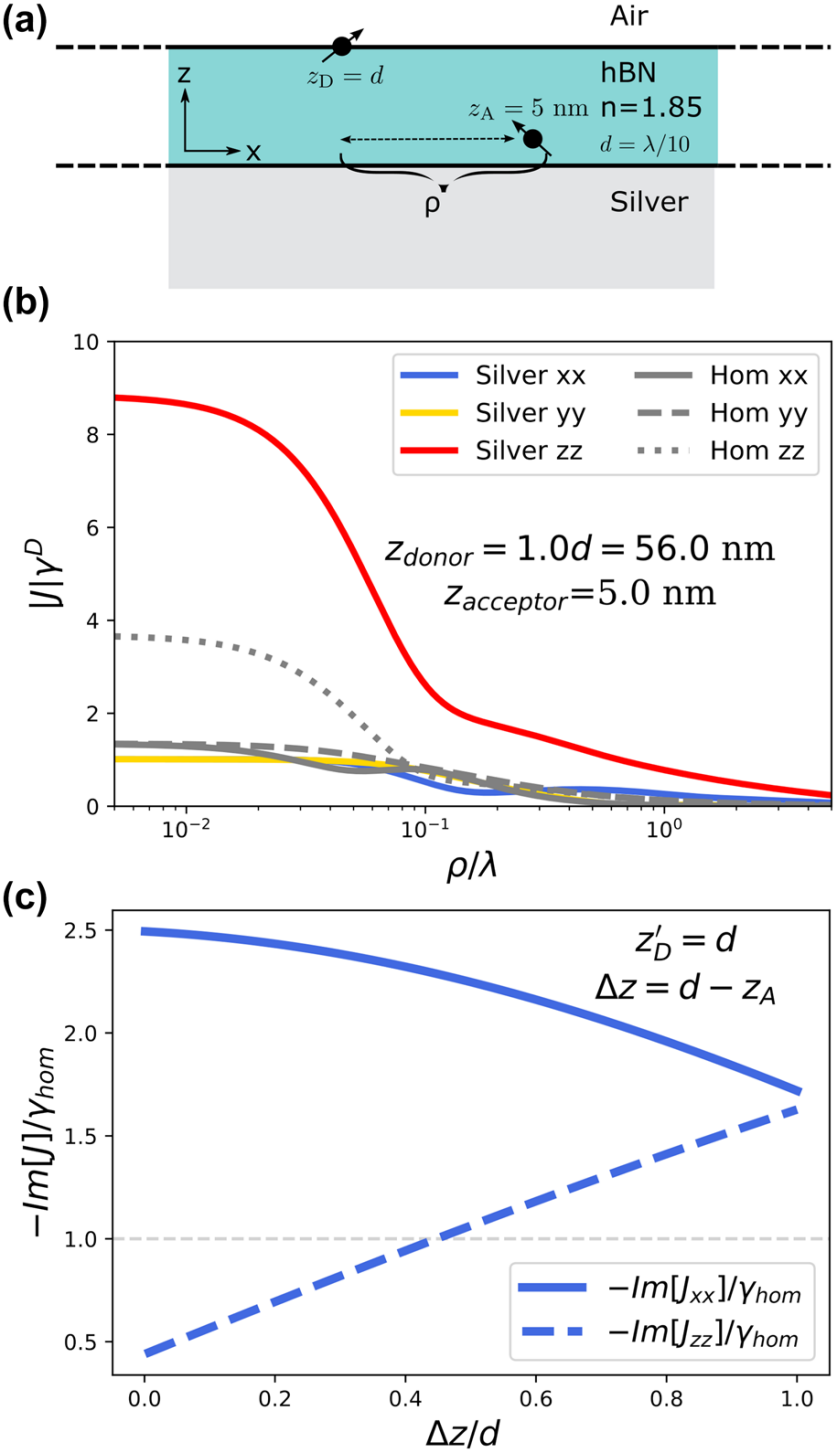
A donor emitter at the upper interface, furthest from the metallic substrate, transfers a photon to the acceptor located near the hBN/silver interface. The acceptor is strongly coupled to the SPP mode. (a) Diagram of the system. (b) Absolute values of the interaction between the donor and acceptor, relative to the donor decay rate, as a function of lateral separation. (c) Imaginary part of the interaction between the donor and the acceptor as a function of acceptor height, with no in-plane separation. The interaction is normalized by the homogeneous hBN decay rate of a single emitter.

The rate of energy transfer goes as the absolute value squared of the Green’s function of the medium [61], recall Eq. (9). We choose for the donor to be placed at *z* = *d*, at the top of the thin film, while the acceptor is placed *z* = 0.09*d* = 5 nm above the silver interface in the hBN film, see Figure 5(a). These choices could be optimized further, but they will already show quite efficient energy transfer.

Referring back to Figure 2(b), we see that the single-emitter decay rate, or equivalently the PLDOS, is small for out-of-plane emitters that are far from the silver interface, but large for ones that are close. The donor will therefore indeed have a much smaller spontaneous-decay rate than the acceptor. Interestingly, the interaction between the pair will be many times larger than the donor decay rate for a large range of in-plane separations *ρ*, as illustrated in Figure 5(b).

For in-plane emitters, the interaction is mostly unchanged by the layered medium when compared to homogeneous hBN, but for the out-of-plane dipole configuration, the presence of surface plasmon polaritons has two important effects. First, it enhances the donor-acceptor interaction, which leads to a greatly enhanced FRET rate. Second, it leads to acceptor decay rates being much larger than the donor decay rates, which makes the energy transfer efficient and one-way. The combination of both effects makes energy transfer in these plasmonic multilayers viable for larger distances than the usual Förster range of ≈10 nm, see also Refs. [74], [75] for alternative schemes.

As seen in Figure 5(b), the FRET rate is highly sensitive to the in-plane separation *ρ*. The maximal FRET rate and efficiency are found for *ρ* = 0, i.e. when donor and acceptor share the same (*x*, *y*) coordinates. The question is whether this maximal FRET rate also gives a good FRET efficiency. To answer this, we will calculate the efficiency using our Eq. (10) and compare with the fundamental efficiency bound *η*_max_ = Γ_*A*_/(Γ_*A*_ + Γ_*D*_) that was derived in Ref. [76]. Until now we have not specified our donor and acceptor spectra. For a fair comparison, we assume to have the same spectral overlap of two Lorentzian distributions as in Ref. [76] between the lifetime-limited donor and acceptor emitters, and we made use of Eq. (7) of Ref. [76] to compute this. In doing so, we find a FRET efficiency for the out-of-plane oriented emitters of *η*_FRET_ = 0.79. This is quite close to the fundamental efficiency bound, which is *η*_max_ = 0.97 (or *η*_max_ = 0.92 when neglecting loss in silver). Our FRET efficiency is also high compared to some of the configurations considered in Ref. [76].

This system is a remarkable case where the imaginary part of the interaction *J*_12_ between the two emitters is larger than the spontaneous-decay rate of the donor. Or equivalently, the PCDOS is larger than the PLDOS at the donor position. The imaginary part of the interaction is shown in Figure 5(c) as a function of the difference in emitter heights within the hBN layer, Δ*z* = *z*_*D*_ − *z*_*A*_ = *d* − *z*_*A*_. At Δ*z*/*d* = 0, the PCDOS coincides with the PLDOS at the position of the donor, while the PCDOS between the donor and acceptor emitters for *ρ* = 0 in Figure 5(b) corresponds to Δ*z*/*d* ≈ 0.9. Notably, for the *z*-oriented dipoles, the PCDOS is larger than the PLDOS at the donor position *z*_*D*_ for all vertical donor-acceptor separations in the hBN layer.

The excited donor emitter will more likely transfer its excitation to the acceptor, with the assistance of the SPP mode, than to decay via single-emitter spontaneous decay. Once the energy is transferred, the acceptor will likely decay spontaneously, into either heat or into an SPP. The latter could then either be used further in a device, or be detected, for example by converting the SPP into a far-field photon [71].

## 4 Discussion: cross density of optical states

The cross density of optical states (CDOS) was introduced in Ref. [77] and characterizes the intrinsic spatial coherence of complex photonic or plasmonic systems. The partial CDOS (PCDOS), defined in Eq. (7), was originally introduced in Ref. [18], and controls the (interference term in the) power emitted by two dipoles [56]. As we have seen, the PCDOS is a useful quantity to understand two-emitter collective emission rates. The same quantity emerges in other contexts, for example in macroscopic quantum electrodynamics as spatial projections of commutators of field operators [78]. The PCDOS has been interpreted as a measure of the number of electromagnetic modes connecting two positions (and orientations), per energy [18], [19], [56]. While we do not question the importance of the concept of the (partial) cross density of states, our present study makes us wonder whether the names “cross density of states” or “cross density of optical states” are appropriate, because of the word ‘density’. We will explain this below.

When we introduced the PCDOS in Section 2, we did give the interpretation that it counts the number of eigenmodes connecting two positions (and orientations) for each energy [56]. And number counting indeed suggests that the concept is related to a density. However, there are important differences between the local density of optical states (LDOS) and the cross density of optical states.

The PLDOS in Eq. (3) is defined as a sum of non-negative terms, one term for each optical mode. However, there is no corresponding property for the PCDOS in Eq. (8), since each term in the mode expansion of the PCDOS can be either positive or negative, so cancellations are possible. It is this type of cancellation of the contributions from different modes that we saw in the layered medium, displayed in Figure 3(c and d). So, whereas contributions from individual optical modes are positive and always add up in the PLDOS, the corresponding terms in the PCDOS can have either sign and may cancel. As an effect of all modes combined, the PCDOS can switch from positive to negative and back as we vary the distance between emitters, as we saw in Figure 3.

Another challenge for the interpretation of the PCDOS as a density or number of modes arose in Figure 5 where the PCDOS connecting two points **r**_*D*_ and **r**_*A*_ turned out to be larger than the PLDOS evaluated at **r**_*D*_. This complicates the notion that the PCDOS counts a number of modes. For how can there be a larger number of modes connecting two distinct points than the number of modes that pass through just a single one of those points? At least the intuitive interpretation does not hold that one starts with the PLDOS at point A and then may lose (but not win) a few modes in the PCDOS along the way when moving one of the two positions from point A to B.

The *mode connectivity* is another useful quantity, defined as the PCDOS normalized by the square root of the product of the PLDOS at its two positions [18]. It does have the interesting property that the mode connectivity of a point A with itself is always unity and larger than the mode connectivity between point A and an arbitrary other point B. However, the mode connectivity is not a density either.

To summarize this discussion, the PCDOS is an important concept that controls two-atom collective emission and has several other uses. However, the ‘D’ in the acronym stands for ‘density’ and here we gave two arguments why this name may give a wrong impression.

## 5 Conclusions

Knowledge in the nanophotonics community how to engineer single-emitter decay rates is well-developed, while much less is known what nanophotonic environments are to be preferred to enhance collective emission. We have explored this for plasmonic and dielectric layered geometries.

We quantified the spectral shifts and collective decay rates of emitters in the experimentally relevant systems of hBN thin films that are freely suspended or placed on a silver substrate. In our group we study quantum emitters in hBN flakes [31], [34] and 2D materials on top of plasmonic surfaces [79]. Currently we are not yet able to create nearby quantum emitters in hBN with (almost) identical emission frequencies, but that may change in the future, perhaps enabling collective emission of the type that we studied here theoretically.

Here we isolated the contributions of the guided modes (including surface plasmons) in single- and multi-emitter decay, and find them to be highly important in mediating the emitter-emitter interaction, even at small emitter separations. The resulting collective decay rates for symmetrically placed emitters in both dielectric and plasmonic systems have sweet spots at finite emitter separation that exhibit a much greater degree of cooperative decay and a longer range than that found in homogeneous media. By ‘sweet spots’ we mean the scaled in-plane distances *ρ*/*λ* in Figure 4 where the damped oscillatory collective decay rates for planar systems exhibit both maxima on the one curve and minima on the other. The amplitudes of these damped oscillations are much larger than in homogeneous media at similar distances. For our planar geometries we find collective decay rates that differ by 40 − 50 % or more from the single-emitter decay rates at emitter separations on the order of hundreds of nanometers.

We found a strong orientation dependence of both single and collective decay rates. By our definitions, the *x*-direction points along the line joining the two emitters, the *y*-direction is the in-plane direction perpendicular to *x*, and *z* points out of plane. For single emitters, rates are strongest modulated for in-plane dipoles in the absence of the metal. With the metal substrate present, perpendicular dipoles point along surface-plasmon field orientations, causing the known strong plasmonic emission-rate enhancement.

Surprisingly, as a result of destructive interference between the guided and the radiative decay channels, the first super/subradiant peak at finite distances is weaker than the second one for the plasmon-assisted *xx*-configuration. While plasmonic systems are often used to enhance light–matter interaction, we find that sometimes the SPP mode interferes destructively with the non-guided part of the inter-emitter interaction, on a distance that is important for collective emission in layered geometries. Similar destructive interference does not occur when considering collective emission by distant identical emitters in 1D waveguides, where the interaction is essentially controlled by the resonant waveguide modes [27]. We do find such destructive interference, which signifies that the cross density of optical states cannot be interpreted as a density.

When combined with life-time limited emitters in hBN, these are promising platforms for realizing collective emission and the technologies that depend upon the collective nature of emission from multi-emitter systems. On the other hand, if emission from single emitters is desired, one should take care to suppress collective emission.

We hope that this work can contribute to the realization of single-photon superradiance in thin films. In the future we could take nonlocal response into account [80]. Furthermore, we treated hBN as an isotropic medium, while it is actually anisotropic due its layered van der Waals nature [64]. A future study could incorporate this anisotropy in the Green’s function using a dyadic permittivity [81].

We also examined energy transfer between two asymmetrically placed emitters within hBN, vertically aligned above a metal substrate. This geometry turns out to give very efficient energy transfer, mediated by propagating surface plasmon polaritons. The energy transfer efficiency is close to a theoretical upper bound. After the transfer to the acceptor, the photon either turns into a single surface-plasmon polariton, or into heat. The SPP could be converted into a single photon in a dielectric waveguide, although 1D waveguides may be more ideal for such conversions than our layered geometries.

More generally, collective emission depends strongly on the dimensionality of the embedding dielectric environment, either in bulk, or in planar or linear waveguides. Here we have investigated planar structures in more detail, and found destructive interference of guided and other radiative modes in the cross density of optical states that determines collective emission rates.

## Figures and Tables

**Figure B.1:**
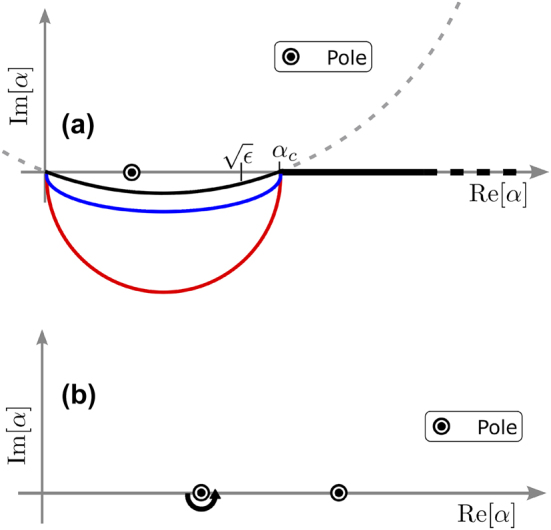
Integration path used to deal with the singularities of poles and the branch points corresponding to $\alpha =\sqrt{\epsilon_{l}}$ . (a) Shown are three different deformed contours that extend into the negative imaginary *α*-plane. The red contour is a semi-circle, the blue is a semi-ellipse and the black is a circle-arc, which the grey dashes showing part of the outline of the bigger circle. After the deformed contour circumvents all problematic points, the integration continues along the real *α*-axis as usual. (b) Contribution from a single guided mode via small contour integral.

## Appendix

### A Multilayer Green function and three-layer system

The response of a medium is quantified by the dielectric function, *ϵ*(**r**). For a layered medium, the dielectric function is piecewise constant, changing only as a function of *z* at the interfaces between different layers, recall Figure 1.

Each layer is characterized only by its dielectric constant and its thickness, *d*_*l*_. Below we briefly recap the properties of the Green function of such layered systems. The Green’s function for a layered medium can be found in various ways. We will make use of the form and notation presented by Tomaš in Ref. [82]. This approach uses the homogeneous Green’s function in the Weyl representation to find the source field of the emitter embedded in layer *j* to then find the scattered field in every layer, from which the fully retarded Green’s function of the layered medium is extracted. While usually only single-emitter decay rates are calculated with this formalism, we will determine collective emission rates. Alternative approaches can be found in Refs. [49], [61], [83], [84], [85]. Using an integral identity of the angular integration, the Green’s function for a three-layer medium is given by [85].

(11)
$${\overset{⃡}{G}}_{lj}\left(r,\omega \right)=\frac{1}{2\pi }\overset{\infty }{\underset{0}{\int }}\text{d}k\;k\left\langle {\overset{⃡}{G}}_{lj}\left(k,\omega ,lz,jz_{0};\rho ,\theta \right)\right\rangle ,$$

where $r=\rho \hat{\rho }+z\hat{z}$ , *k* is the magnitude of the in-plane component of the wavevector and *l* and *j* represent the layers of the final and initial positions, respectively. The notation *jz*_0_ means that the *z*_0_-coordinate is taken with respect to the bottom of the *j*’th layer, and similarly for *lz*. For the bottom layer, the *z*-coordinate would be negative.

For our purposes of color centers within the same thin layer, we only need to consider the Green’s function connecting two points within the central layer of a three-layer medium. With **r** and **r**′ both located in layer *l* = *j* = 2, and assuming that *z* > *z*_0_ (where a finite *z*-difference is required for convergence, see Appendix C), the angle-averaged Green’s function in the Weyl representation is given by

(12)
$$\begin{array}{cc} & \left\langle {\overset{⃡}{G}}_{22}\left(k,\omega ,z,z_{0};\rho ,\theta \right)\right\rangle \\ & \;=-\frac{i}{2\beta_{2}}\underset{q=\text{TE,TM}}{\sum }\frac{\xi_{q}}{D_{2}^{q}}\left[\left\langle {\hat{e}}_{q2}^{+}\left(k\right){\hat{e}}_{q2}^{-}\left(-k\right)\right\rangle {\text{e}}^{\text{i}\beta_{2}\left(z-z_{0}\right)}\right.\\ & \;+r_{23}^{q}\left\langle {\hat{e}}_{q2}^{-}\left(k\right){\hat{e}}_{q2}^{-}\left(-k\right)\right\rangle {\text{e}}^{\text{i}\beta_{2}\left(-z-z_{0}+2d\right)}\\ & \;+r_{21}^{q}\left\langle {\hat{e}}_{q2}^{+}\left(k\right){\hat{e}}_{q2}^{+}\left(-k\right)\right\rangle {\text{e}}^{\text{i}\beta_{2}\left(z+z_{0}\right)}\\ & \;+\left.r_{23}^{q}r_{21}^{q}\left\langle {\hat{e}}_{q2}^{-}\left(k\right){\hat{e}}_{q2}^{+}\left(-k\right)\right\rangle {\text{e}}^{\text{i}\beta_{2}\left(-z+z_{0}+2d\right)}\right]. \end{array}$$

The Green’s function for *z* < *z*_0_ can be found through interchange of **r** and **r**′. In Eq. (12), the *ξ*_TM_ = 1 and *ξ*_TE_ = −1 are polarization-dependent sign factors, $\beta_{l}\equiv \sqrt{k_{l}^{2}-k^{2}}$ is the pseudo-*z*-component of the wavevector, and the $r_{ij}^{q}$ are the Fresnel reflection coefficients for *q*-polarization between layers *i* and *j*. For the angle-averaged dyadic outer products of the polarization vectors in Eq. (12), such as $\left\langle {\hat{e}}_{\text{TE}l}^{\pm }\left(k\right){\hat{e}}_{\text{TE}j}^{\pm }\left(-k\right)\right\rangle$ , we refer to Eq. (B.8) in Appendix B of Ref. [85].

#### A.1 Guided modes and the deformed contour integral

Of special note in Eq. (12) for the multilayer Green function is the multiple-scattering parameter.

(13)
$$D_{l}^{q}=1-r_{l-}^{q}r_{l+}^{q}e^{2i\beta_{l}d_{l}},$$

where $r_{l+}^{q}$ and $r_{l+}^{q}$ are the total reflection coefficients of the stacks above and below layer *l*. In the form $1/D_{l}^{q}$ , it accounts for the infinite (geometric) series of reflection events of all orders in the central layer and the phase gained from traversing the *l*’th layer twice. This can be seen by carrying out the Taylor expansion $\frac{1}{1-x}=\sum_{n=0}^{\infty }x^{n}$ . This is true for purely dielectric systems, where the Taylor expansion of $1/D_{l}^{q}$ affords a very pictorial description of this multiple-scattering mechanism, but also for metallic systems that can carry SPP modes.

In general, the zeroes of $D_{l}^{q}$ correspond to guided modes in the *l*’th layer. If we let the thickness of the hBN layer tend to zero, then we find a zero of the multiple-scattering parameter for TM-polarized light that corresponds to the textbook surface plasmon polariton dispersion $k_{\text{SPP}}=k_{0}{\left[\epsilon_{d}\epsilon_{m}/\left(\epsilon_{d}+\epsilon_{m}\right)\right]}^{\frac{1}{2}}$ for a single dielectric-metal-interface [86], as it should. No analogous surface-plasmon condition is found for the TE modes, as is well-known for single-interface surface plasmons. Nevertheless, increasing the thickness of the hBN layer will eventually allow for multiple guided modes to form, some of which can be TE polarized. These larger thicknesses are outside the scope of the present paper.

In general the mode index, i.e. the value of *α* = *k*/*k*_0_ for which we have guided modes, has to be found through numerical solution of the condition $D_{l}^{q}=0$ , at the relevant wavelength of light of interest (here *λ* = 560 nm). For lossless systems, this results in real-valued *α*, while with loss, the poles migrate into the upper half of the complex *α*-plane, as these modes correspond to exponentially damped waves [85]. For lossless media it is possible to isolate the contribution of the guided modes that appear on the real axis by evaluating the integral of a small open contour tightly surrounding the pole. Therefore, we will sometimes consider a “lossless metal” to isolate the contributions of the guided modes with metallic substrates. This is a useful an accurate approximation for our purposes since finite propagation lengths of SPPs are usually much longer than typical distances between emitters that send out light collectively. For more details on this approximation we refer to Appendix B. Ref. [87] provides a means of counting guided modes for three-layer lossless dielectric systems. In fact, it was shown that for a symmetric three-layer system with lossless dielectric materials, there will always be at least one TE- and one TM-polarized guided mode.

For guided modes in a lossless system, the residue from the contour integral around the pole is purely real-valued, making the guided-mode contribution to the Green’s function purely imaginary via the residue theorem. From Eqs. (5), (6), and (7), we see that this means that the contribution of the guided modes becomes evident only in the decay rates of single or multiple-emitter systems. In this work we do not consider each guided mode separately, but instead include all the guided-mode contributions together.

### B Contour integration

In order to take into account the contributions of guided modes, which are represented by poles of the Green’s function integrand, we partition the integration in Eq. (11) into two parts. In the first, we utilize a deformed integration contour, that goes from 0 to a cutoff value *α*_c_, and then dips into the negative complex plane. This cutoff value has to be large enough that all poles on the real axis have been circumvented. For examples and details, see Refs. [49], [85], [88], [89], [90]. The second integral is then performed from *α*_c_ to infinity along the real axis. While a semi-circle path is easy to implement, going far into the fourth quadrant causes exponential decay. A semi-elliptical contour is often recommended in order to better control the depth into the imaginary plane [85], [89]. To avoid the large curvatures near the edges of the integration path when the eccentricity of the ellipse is large, we instead make use of a circle arc, shown in black in Figure B.1(a) . In order to isolate the contributions of the guided modes in lossless media, we make a tight semi-circle integration around the pole, as illustrated in Figure B.1(b). Since the pole is located on the real axis, we only pick up the contributions from going half-way around the pole, i.e. only half the residue contributes to the integral. For lossy metals the pole moves into the first quadrant, where branch cuts significantly complicate the process of isolating the contribution from the guided mode [85], [88]. Thick layers can be host to multiple guided modes. If these have very similar mode indices, then it can be difficult to isolate their contributions. The same is true for thin layers, where the mode index of the guided mode tends towards the refractive index of the surrounding medium.

### C Convergence of the contour integration

In order to get the integral in Eq. (11) to converge, we require a finite difference in the *z*-coordinates of the emitters positions, i.e. |*z* − *z*_0_| > 0. This can further be justified by the implicit choice of an exclusion volume, as rigorously studied by Yaghjian [91], for our layered medium. However, we are free to choose a finite, but physically insignificant, value. We chose.

(14)
$$\Delta z=\max\left(\Delta z_{\min},\rho \sin\left(\phi_{\max}\right)\right),$$

such that for small separations along the in-plane direction, the vertical separation is constant and bounded from below by Δ*z*_min_, and for larger in-plane separations the vertical off-set grows with increasing distance. Our choice of lower bound value is Δ*z*_min_ ≈ 0.5 Å, much smaller than other physically relevant length scales. The upper bound is determined by the maximal angle between the actual separation of the points **r** and **r**′ and the projection onto the horizontal plane, for which we used *ϕ*_max_ = 0.05°, leading to a maximal vertical displacement |*z* − *z*_0_| < 1.5 nm for a maximal separation *ρ* = 3*λ* = 1,680 nm.

For the largest distances between the emitters that we consider (three wavelengths), choosing the smallest value of the exclusion volume of 0.5 Å would result in extremely slow convergence of the integral Eq. (11). For smaller distances, we can take the exclusion length smaller without the very slow convergence. With our current choices of exclusion volumes, we do not have this problem of slow convergence, and we checked convergence: our results do not change by making exclusion volumes slightly larger or smaller.
